# Effective long-term treatment with incobotulinumtoxin (Xeomin®) without neutralizing antibody induction: a monocentric, cross-sectional study

**DOI:** 10.1007/s00415-019-09681-7

**Published:** 2020-01-20

**Authors:** Harald Hefter, Raphaela Brauns, Beyza Ürer, Dietmar Rosenthal, Philipp Albrecht

**Affiliations:** Department of Neurology, University of Düsseldorf, Moorenstrasse 5, 40225 Düsseldorf, Germany

**Keywords:** Incobotulinumtoxin, Long-term treatment, Neutralizing antibodies, Low antigenicity, Complex proteins

## Abstract

**Background:**

Among the spectrum of licensed botulinum neurotoxin preparations incobotulinumtoxin (incoBoNT/A; Xeomin®) is the only one which does not contain complex proteins. Therefore, incoBoNT/A has been suggested to have a low antigenicity, but precise estimations on incidence and prevalence of neutralizing antibody formation during long-term treatment are outstanding so far.

**Methods:**

For the present cross-sectional study, 59 patients having exclusively been treated with incoBoNT/A (mono group) and 32 patients having been treated with other BoNT/A preparations less than nine times and who were then switched to at least 14 sessions of incoBoNT/A treatment (switch group) were recruited from one botulinum toxin outpatient clinic. Side effects and doses were extracted from the charts, and the efficacy of treatment was assessed by the patients using a visual analogue scale (0–100). The prevalence of neutralizing antibodies was tested by means of the mouse hemi-diaphragm assay (MHDA).

**Findings:**

None of the patients in the mono and only two in the switch group had a positive MHDA-test. Across all indications and patients, mean improvement exceeded 67%. Improvement did not depend on age at onset, sex, change of dose or duration of treatment, but on disease entity. In patients with cervical dystonia, improvement was about the same in the mono and switch subgroup, but the last dose was different.

**Conclusions:**

The present study confirms the low antigenicity of incoBoNT/A, which has immediate consequences for patient management, and the use of higher doses and shorter durations of reinjection intervals in botulinum toxin therapy.

## Introduction

The popularity of botulinum neurotoxin (BoNT) applications is continuously growing among clinicians and the general public [1]. After the first clinical application by the ophthalmologist Alan Scott, who successfully corrected eye muscle disbalance, BoNT was used to treat focal muscular hyperactivity in the face, head and neck muscles. Meanwhile physicians from diverse specialties are integrating botulinum toxin injections into their practices ranging from the treatment of incontinence, pain, headache, and hyperhidrosis [1] to the reduction of postoperative complications e.g. in cardiac surgery [2]. But the general popularity of BoNT was reached mainly after BoNT was used for cosmetic indications. Botulinum neurotoxin type A (BoNT/A) injections have become the most popular of all cosmetic procedures worldwide [3].

With further increase of the spectrum of indications of BoNT/A applications, the problem of antigenicity of BoNT/A preparations has become increasingly relevant. For most indications, repetitive injections of botulinum neurotoxin have to be performed [4] to maintain a certain level of improvement. Since these repetitive injections are applied transdermally, activation of dentritic cells can hardly be avoided [5] with the risk of neutralizing antibody (NAB) formation. The question remains as to after how many repetitive BoNT injections, clinically relevant antibody titres and secondary reduction of response to therapy occur. For several indications, it has been reported that secondary treatment failure (STF) may occur even after one to three injections [6, 7]. In patients with CD who developed a complete STF later on in the course of treatment, it could be demonstrated that their response to BoNT/A injections was lower from the second injection on than in patients who did not develop an STF [8]. This early reduction of response is probably difficult to detect as long as neither treating physician nor patient expect such a complication of BoNT/A therapy at that time.

Induction of antibodies and the antigenicity of a BoNT preparation depends on the content of a BoNT/A vial. This differs considerably between different BoNT/A preparations [9]. The protein complex being produced by *Clostridium botulinum* does not only contain the 150 KD large neurotoxin type A molecule, but also associated complexing proteins, which after oral uptake protect the BoNT/A molecule during its passage through the acidic milieu of the stomach [10] and allow its transmigration through the intestinal epithelial barrier [11]. There has been a debate whether complex proteins are a help or a hindrance for the BoNT/A molecule when it is injected directly into a tissue bypassing the gastrointestinal tract [12]. Meanwhile it has been demonstrated that the complex proteins rapidly dissociate from the BoNT/A molecule after reconstitution of a vial even prior to injection [13], so that on the one hand the assumed shielding of epitopes [14] against neutralizing antibodies does not take place. On the other hand, the complex proteins (especially the hemagglutinin HA-33) may act as adjuvants enhancing the immune response to a BoNT/A injection [15, 16].

BoNT/A preparations not only differ with regard to complex proteins, but also in the content of albumin and flagillin [9]. Furthermore, the percentage of biologically inactive, but immunologically active BoNT/A molecule fragments is different [1]. In the incoBoNT/A preparation (Xeomin®), the biologically inactive fragments have been removed and the total clostridial protein content of a vial of 100 U is reduced to 0.44 ng [1]. In line with this, animal experiments suggest that the incoBoNT/A preparation has a low antigenicity [17]. However, one has to be cautious when transferring non-primate immunological study results to humans.

Clinical experience was that the “old” formulation of onaBoNT/A (Botox®) had a high protein load and a high antigenicity [18]. Purification and a fivefold reduction of the protein load led to a considerable reduction of the risk to develop antibodies by a factor of 6 [19–21]. The protein content of the incoBoNT/A preparation is even lower than that of the “new” onaBoNT/A preparation (5 ng/vial of 100 U; [1]). Therefore, it has been hypothesized that the antigenicity of incoBoNT/A may be lower than that of abo- or onaBoNT/A. However, this has not been demonstrated so far, in primate animal experiments or in human studies.

To demonstrate the differences in antigenicity between BoNT/A preparations, careful long-term studies are warranted with comparable doses per session, inter-injection intervals, and duration of treatment, since these three factors are the main influence for NAB formation [22, 23]. Furthermore, precise estimations on the incidence and prevalence of NAB formation have to be determined for each BoNT/A formulation. This study aims to determine the incidence and prevalence of NAB formation under incoBoNT/A long-term treatment as well as a confounding effect of preceding injections with a complex protein-containing preparation (abo- or onaBoNT/A). Long-term efficacy is also controlled to demonstrate that the clinical response matches the findings on antigenicity of incoBoNT/A.

## Methods

All patients gave written informed consent and the study was performed according to the guidelines of good clinical practice (GCP) and had been approved by the local ethics committee of the University of Duesseldorf (Germany) in accordance with the Declaration of Helsinki.

### Patients (mono and switch group)

A retrospective chart review of all patients treated at the BoNT outpatient clinic of the Department of Neurology of the University of Düsseldorf (Germany) identified those patients, who had started incoBoNT/A treatment in our department and had been treated uniquely with incoBoNT/A since then.

Inclusion criteria were (1) age older than 18 years, (2) not under legal care, (3) no interruption of incoBoNT/A therapy for longer than 5 months, and (4) written informed consent. Patients with a history of more than eight injections with abo- or onaBoNT/A before they were switched to incoBoNT/A were excluded. Patients with a history of less than 9 abo- or onaBoNT/A injections, but less than 14 following incoBoNT/A injections were also excluded. This criterion was used because of our experience that NAB titres may decrease below the detection level under continuous incoBoNT/A therapy for more than 3 years [24].

Finally, 62 patients were included who had exclusively been treated with incoBoNT/A (mono group) and 33 patients who had received 8 or less previous abo- or onaBoNT/A injections and at least 14 following treatments with incoBoNT/A without interruption (switch group).

In two patients of the mono group, blood samples were lost during the transport, and further two patients (one in the mono and one in the switch group) withdrew written informed consent because they did not want to wait until blood samples were taken. The final analysis is based on 59 patients in the mono and 32 patients in the switch group.

For further analysis, patients were subdivided into a subgroup of patients with facial dystonia (FD; patients with hemifacial spasms or simple blepharospasm; *n* = 9; triangles in the figures), patients with other focal, multifocal or segmental dystonia (ODT; severe Meige syndrome and/or oropharyngeal or oromandibular dystonia; *n* = 7; squares), patients with idiopathic cervical dystonia (CD; *n* = 73; circles), and patients with spasticity after stroke (SPAS; *n* = 2; diamonds).

### Determination of neutralizing antibodies

At the day of recruitment, blood samples were taken and deep frozen until all patients had been included. Then, blood samples were coded and sent off to the Toxogen® laboratory (Hannover, Germany) to be analysed by means of the mouse hemidiaphragm assay (MHDA) for the presence of neutralizing antibodies in one batch. The Toxogen® laboratory had not been informed on the purpose of the study and had not received any clinical data of the patients. The laboratory determined the paralysis times which were the outcome measures of the MHDA [25]. A complete list of paralysis times and whether a blood sample was classified as positive or not was returned to our institution.

### Further outcome measures

For each treatment date of injection, the reported side effects, BoNT/A preparation used, and each single dose were extracted from patients’ charts. From the charts of CD patients, also the TSUI score (estimating the severity of CD [26]) which had been determined and documented by the treating physician before each treatment was extracted. On the day of inclusion, patients were asked to rate their subjective improvement of symptom severity (IMP) in percent of the severity before initiation of BoNT therapy. In CD patients the last TSUI score (LTSUI) was determined at inclusion.

### Statistics

Overall improvement (IMP), treatment-related, demographical and safety data were reported as mean values and standard deviations, or absolute numbers or percentage where appropiate. Student’s *t* test and non-parametric correlation analysis (rank correlation) were used to analyse the influence of age at onset of therapy, sex, duration of treatment, initial dose of incoBoNT/A, and increase of dose on improvement (IMP). For some parameter combinations, also a regression line and the Pearson correlation coefficient were calculated. All tests used were part of the SPSS statistics package (version 25; IBM, Armonk, USA).

## Results

### Demographical data, side effects, and treatment-related data

In Table 1, the demographic data of the entire cohort, the mono and the switch subgroup are presented. Age at onset and sex distribution were comparable in these three groups.

**Table 1:** Demographic, treatment-related data and outcome

Parameter	MV (all patients)	SD	MV (mono)	SD	MV (switch)	SD	Significance level
Age at onset of therapy	52.9	12.5	53.1	13.3	52.5	11.2	n.s.
Female/male ratio	53/38		35/24		18/14		n.s.
Duration of treatment (days)	2149	1225	1646	944	3074	1150	*p* < 0.001
Initial dose (U)	193	93	184	76	210	118	n.s.
Last dose (U)	278	109	261	92	311	130	*p* < 0.033
Improvement (IMP, %)	67.9	22.9	70.2	22.2	61.0	23.3	*p* < 0.030
Initial TSUI (ITSUI) in CD patients only	8.3	3.5	7.9	3.5	9	3.6	n.s.
Last TSUI (LTSUI) in CD patients only	3.9	2.6	3.6	2.4	4.5	2.8	n.s.

*MV* mean value, *SD* standard deviation, *n.s.* not significant

No severe treatment-related side effects had been documented, requiring special treatment or hospitalization. The number of side effects was highest in the ODT subgroup (> 10%/injection cycle) because of the difficulty in injecting the muscles of the mouth, jaw, and throat. Patients claimed on dysarthria, difficulties in swallowing and/or biting hard pieces of food. No side effects were reported in the small SPAS subgroup. The most frequently reported side effect during the first year of treatment in the FD subgroup was transient double vision and ptosis (5%/injection cycle). In the CD subgroup, weakness and dysarthria were reported in less than 3%/injection cycle. The number of side effects/injection cycle decreased with the duration of treatment.

**Fig. 1:**
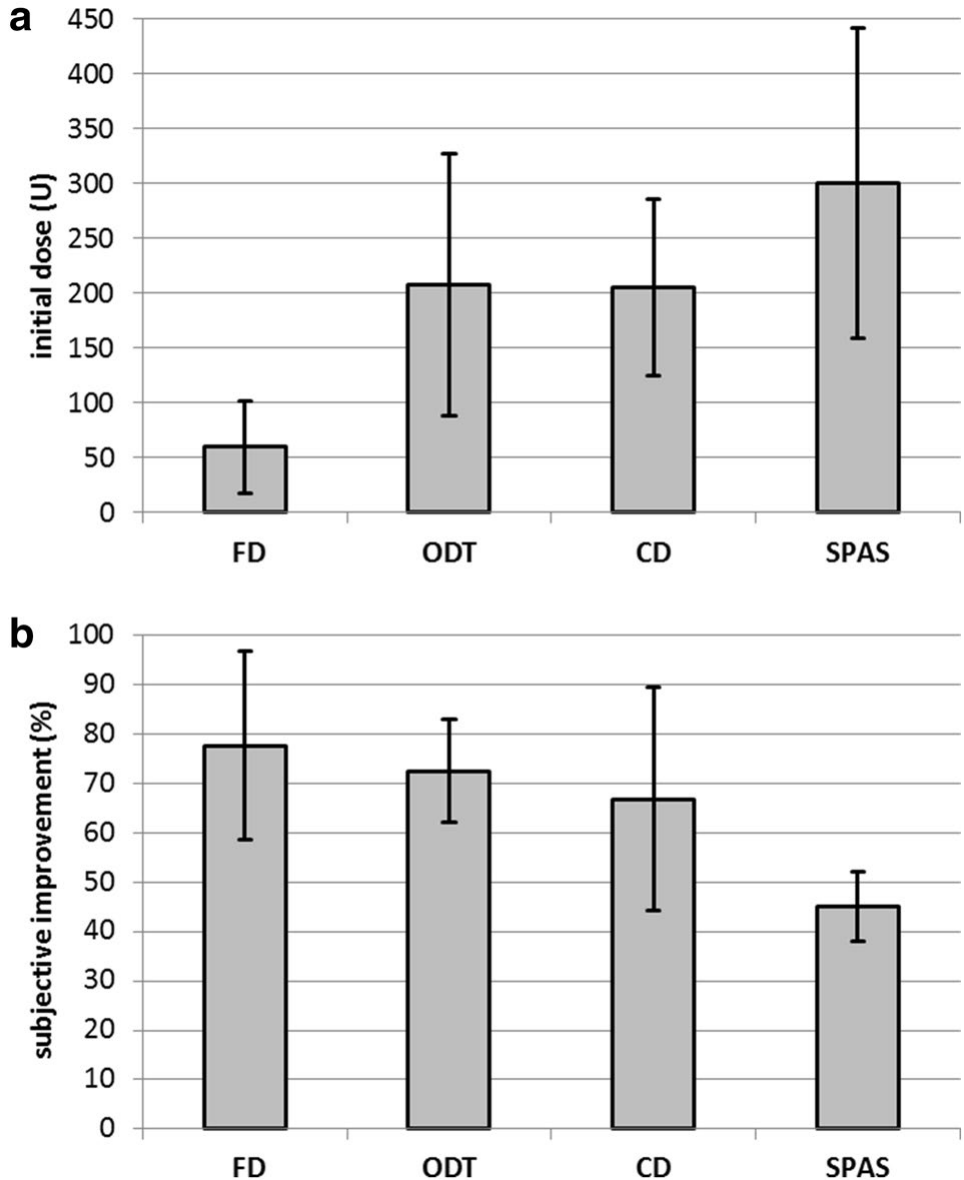
**a** Mean initial dose of incoBoNT/A and standard deviation of the FD, ODT, CD, and SPAS subgroup (for definition of these subgroups, see “Methods”). **b** Mean subjective improvement and standard deviation of the FD, ODT, CD and SPAS subgroups

The initial dose of incoBoNT/A was 193 ± 93 U in the entire cohort. In the switch group, the initial dose (210 ± 118 U) was only slightly higher than in the mono group (184 ± 76 U). The initial dose per session was obviously different for different disease entities, and lowest in the FD and highest in the SPAS subgroup (see Fig. 1a). During the course of treatment, incoBoNT/A doses per session were significantly increased (*r* = 0.219; *p* < 0.038) by about 85 MU in the entire cohort. In the mono group, the dose was increased by 77 MU and in the switchers by 101 MU in the mean, resulting in a significant (*p* < 0.033) difference of the last dose between the mono (261 ± 92 U) and the switch group (311 ± 130 U). For most of the switchers, dose per session of abo- or onaBoNT/A treatment was not available.

The treatment interval of more than 95% of the patients was either 12 or 13 weeks with a mean interval between injections of 87 ± 5.2 days. The mean duration of treatment was 2149 ± 1225 days in the entire cohort corresponding to 6.0 years of treatment and more than 24 injections. In the switch group, the mean duration of treatment of treatment was 3074 ± 1150 days including a duration of pre-treatment with abo- or onaBoNT/A of 547 ± 95 days. The duration of treatment with incoBoNT/A was 2527 ± 1020 days in the switch and 1646 ± 944 days in the mono group. The mean duration of incoBoNT/A treatment in the entire group was 1956 ± 986 days corresponding to more than 5.3 years of incoBoNT/A treatment and application of more than 22 injections of incoBoNT/A.

### Antibody formation under incoBoNT/A treatment

The primary outcome measure of the present study was the result of the MHDA. In all patients of the mono group, the MHDA was negative, and the paralysis time was well below the cutoff level for a positive test result (61 min), which had been established by the Toxogen®-Lab. The prevalence and incidence of NAB formation in the mono group were zero.

In the switch group, two patients with CD had a positive MHDA test. In both patients, the paralysis time was beyond the upper time limit of the MHDA (> 130 min), corresponding to very high antibody titres. Both patients had been pre-treated with aboBoNT/A. The prevalence of NABs in the switch group was 6.3%, and the estimation of mean NAB incidence was less than 0.75%/year (= 6.3%/8.5 years).

In the entire cohort, NAB prevalence was 2.2%, and estimation of NAB incidence was 0.37%/year. Under the assumption that NABs had been induced during the incoBoNT/A treatment in the two MHDA-positive patients, “worst case” estimation yields a mean incidence of NAB formation under incoBoNT/A treatment of 0.41%.

### Efficacy of incoBoNT/A treatment

**Fig. 2:**
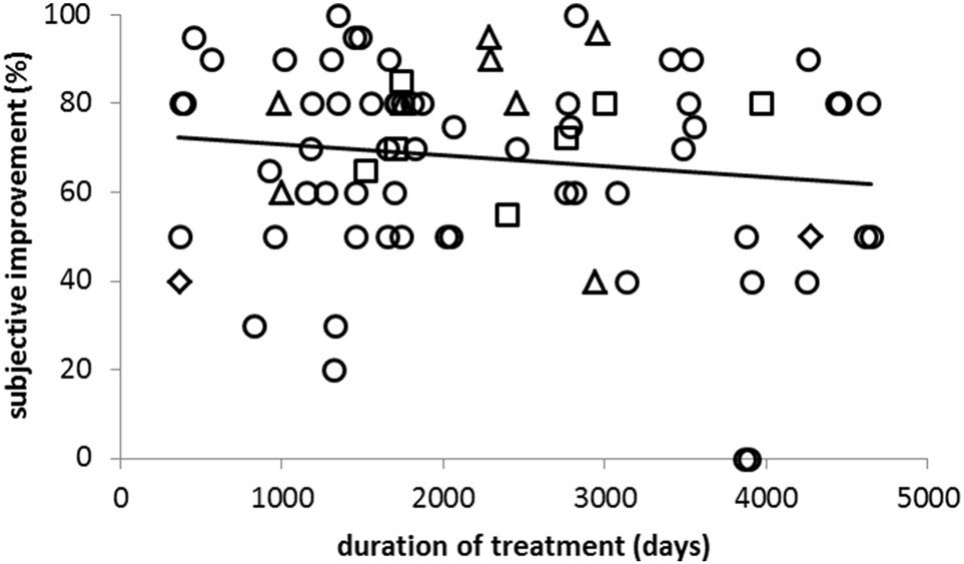
Subjective improvement (IMP; *y *axis) does not correlate (*r* = − 0.139; n.s.) with the duration of treatment of incoBoNT/A treatment (*x* axis). (triangles, patients with FD; squares, patients with ODT, circles, patients with CD, diamonds, patients with SPAS; for definition of the FD-, ODT-, CD- and SPAS subgroup see “Methods”)

**Fig. 3:**
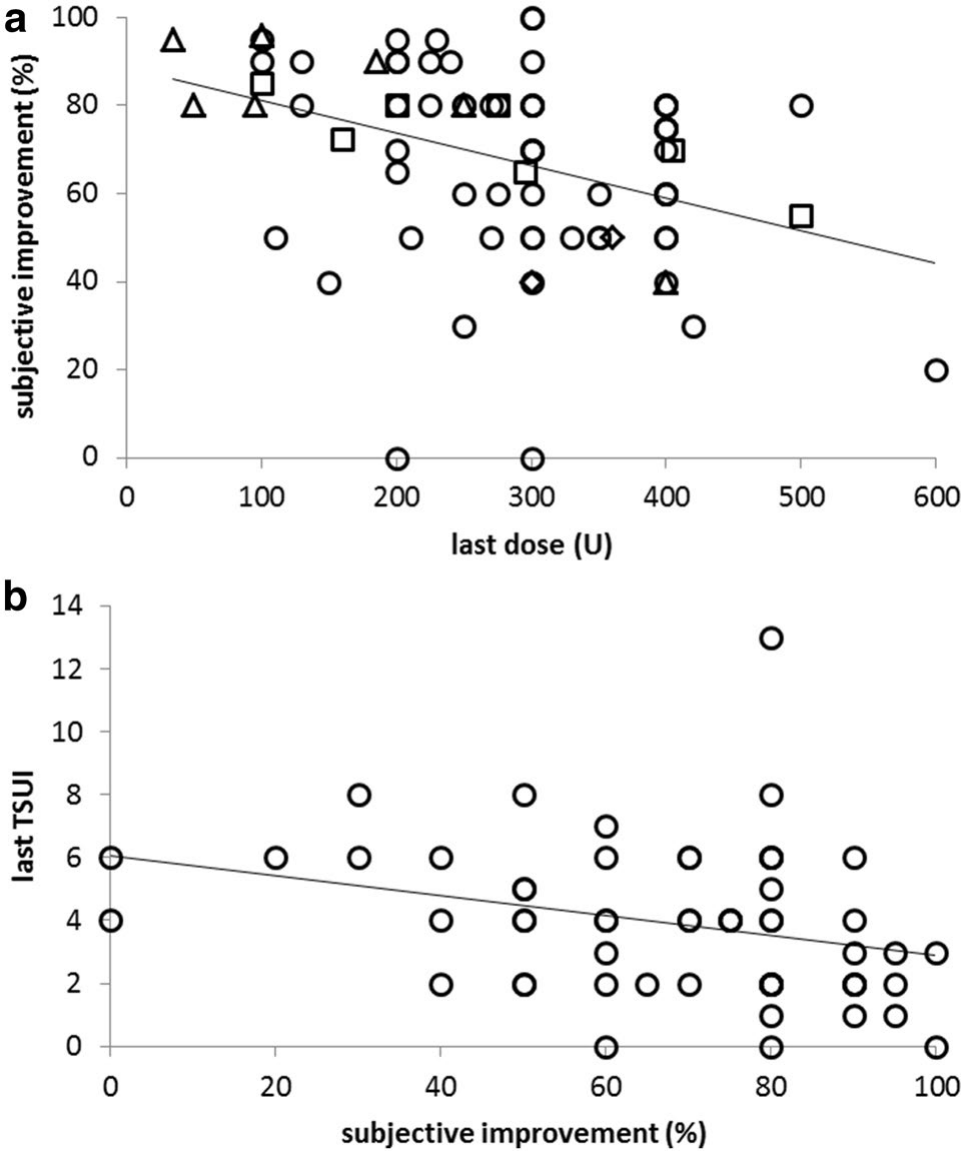
**a** There is a significant negative correlation (*r* = − 0.450; *p* < 0.001) between subjective improvement (IMP) and last dose. (triangles, patients with FD; squares, patients with ODT, circles, patients with CD, diamonds, patients with SPAS; for definition of the FD, ODT, CD and SPAS subgroup; see “Methods”). **b** In patients with cervical dystonia there is a significant correlation (*r* = − 0.303; *p* < 0.021) between last TSUI score and subjective improvement

The (secondary) clinical outcome measure was subjective improvement (or worsening) in % of the severity of the disease before onset of incoBoNT/A therapy (IMP). The mean IMP was 67.9% in the entire cohort. It differed between disease entities (Fig. 1b) and was highest in the FD (77%) and lowest in the SPAS subgroup (45%). Despite the side effects IMP was high (72%) in the ODT subgroup (see Fig. 1b). Neither age at onset, nor sex, nor change of dose had any effect on IMP. No correlation between IMP and duration of treatment was found (Fig. 2). In the entire cohort, there was a significant negative correlation (− 0.0737 × (last dose) + 88.6; *r* = − 0.45, *p* < 0.001) between IMP and last dose (Fig. 3a).

### Efficacy of incoBoNT/A treatment in patients with CD

IMP in the CD patients was 66.8% in the mean and significantly (*p* < 0.03) higher in the mono (> 70%) than in the switch group (61%). In patients with cervical dystonia, the TSUI score was used as a further outcome measure. The last TSUI score (LTSUI) was 3.9 in the mean. In the mono group, the mean LTSUI was even lower (3.6) than in the switch group (4.5; see Table 1). The TSUI at the time of switch to incoBoNT/A was not significantly higher in the switch compared to the mono group. Also, the last TSUI did not differ significantly between the mono and switch group (see Table 1). The intercept and the steepness of the regression line between the last dose and last TSUI (LTSUI = 0.0109 × (last dose) + 0.73; *r* = 0.415; *p* < 0.001) were lower than these values of the regression line between the initial dose and initial TSUI (ITSUI = 0.0219 × (initial dose) + 2.28; *r* = 0.444; *p* < 0.001). TSUI score before and after incoBoNT/A treatment did not correlate (*r* = 0.156; n.s.). IMP was significantly negatively correlated with LTSUI (*r* = − 0.303; *p* < 0.021; Fig. 3b) and positively with the improvement of the TSUI score (ITSUI–LTSUI; *r* = 0.229; *p* < 0.031).

### Temporal development of TSUI scores and doses per session in the mono and switch group and in the two MHDA test-positive patients

**Fig. 4:**
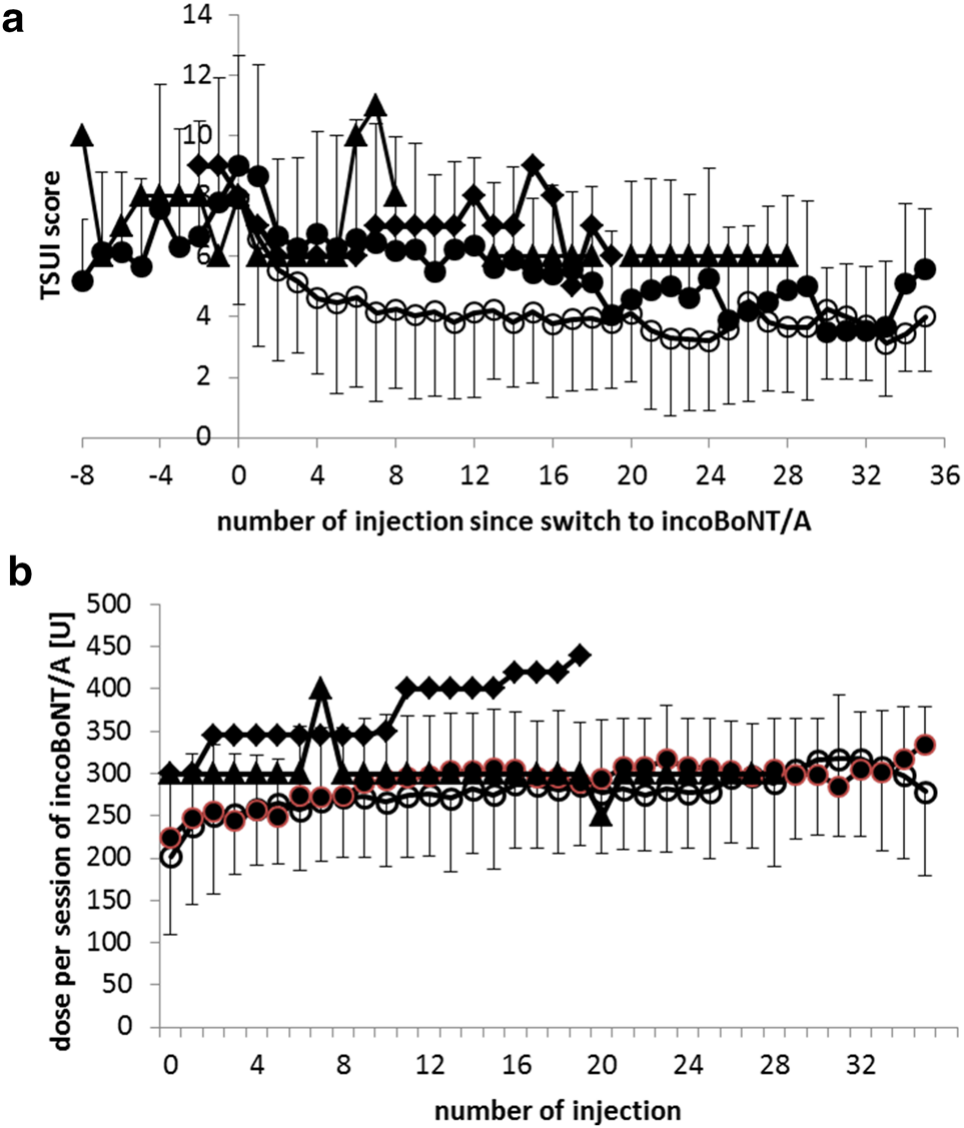
**a** Comparison of the temporal development of mean TSUI scores and standard deviations in the mono (open circles) and in MHDA-negative CD patients in the switch group (full circles). Two MHDA-positive switchers (full triangles and diamonds resp.) were presented separately. **b** Comparison of the temporal development of mean single incoBoNT/A doses per session and standard deviations in the mono (open circles) and in MHDA-negative CD patients in the switch group (full circles). Two MHDA-positive switchers (full triangles and diamonds resp.) were presented separately

In Fig. 4a, the temporal development of the mean TSUI score in the mono (open circles) and MHDA-negative CD patients in the switch group (full circles) is presented. During the first few treatment cycles, switchers responded less well in comparison to the patients in the mono group, but the final outcome after a few years did not differ significantly. The mean doses per session (Fig. 4b) did not differ significantly at onset of incoBoNT/A-therapy, but the last dose was significantly (*p* < 0.033) larger in the switch group (see Table 1).

The first MHDA-positive patient (age at onset: 52.3 years; female; full triangles in Fig. 4) had been treated with 4 aboBoNT/A and 4 incoBoNT/A injections (with 720U, 720U aboBoNT/A, 300 U, 250 U, 300 U, 300 U incoBoNT/A, 880 U and 880 aboBoNT/A) every 3 months before the final switch to incoBoNT/A. The second MHDA-positive patient (age at onset: 69.2 years; female; full diamonds in Fig. 4) had been pre-treated with only 2 aboBoNT/A injections (880 U and 880 U). No booster injections had been performed. Both patients did not respond well (Fig. 4a) to abo- or to incoBoNT/A, although sufficiently high doses had been used (Fig. 4b).

## Discussion

### Long-term treatment with incoBoNT/A is safe and effective

Analysis of safety aspects were not the primary focus of the present study. However, it is worth mentioning that no treatment-related severe adverse event had been documented in the charts. Mild to moderate severe events either resulted from the injection of small muscles as in the FD subgroup or from the treatment of muscles that are difficult to inject as in the ODT subgroup. Overall, side effects were observed in less than 5% of the patients per injection cycle, which is in the order of side effects usually observed in open studies and well below the percentage reported in double-blind studies on incoBoNT/A (e.g. [27]).

Subjective improvement of severity of symptoms (IMP) was the secondary outcome measure of this study. IMP was clearly dependent on disease entity (see Fig. 1b). In the FD group, which was easiest to inject and received the lowest doses, IMP was the highest (> 75%), while in the higher dose spasticity subgroup (SPAS) IMP was the lowest (< 50%). In the difficult to inject ODT subgroup, IMP was surprisingly high (72.5%). Obviously, the severity and frequency of experienced side effects did not impact the patients’ rating of an effective treatment.

In the large (*n* = 73) CD subgroup, the mean IMP was 66.8%, which is higher than the 53.4% reported in a previous study on long-term treatment of CD [22, 28]. In this study, IMP was highly significantly (*p* < 0.003) correlated with all sub-scores of the CDQ24 questionnaire (analysing various aspects of quality of life as stigma, emotional well-being, pain, everyday life activities, social life; for details see [28]) and with treating physician’s rating (*p* < 0.001; [28]). This matches the significant correlation between IMP and the last TSUI score and between IMP and the improvement of the TSUI score in the present study. In the previous study [28], the improvement rated by the treating physician did not correlate with the sub-score emotional well-being. Thus, rating of the improvement by the patient takes into account consequences of disease, which cannot be scored by the treating physician.

In the present study, the remaining severity of CD after long-term incoBoNT/A treatment evaluated by means of the TSUI score was lower in the mono group (3.6 ± 2.4) than in the switch subgroup (4.5 ± 2.8), which is very close to the value of 4.75 previously reported for an antibody Elisa test-negative subgroup of CD patients after BoNT/A long-term treatment [22]. This confirms the efficacy of long-term treatment with incoBoNT/A.

Initial doses were in the order of doses used in other studies on treatment with incoBoNT/A (see Table 1 and [27]). Doses were significantly increased with duration of treatment, which is also in line with previous reports on long-term treatment with BoNT/A (e.g. [29]).

### The risk to induce NAB is low during long-term treatment with incoBoNT/A

This is the first time that the incidence and prevalence were determined in a larger cohort of patients who had exclusively been treated with incoBoNT/A over a longer time period. In the mono group, none of the patients had a positive MHDA test. This is in complete agreement with the observation of others that so far no patient having exclusively been treated with incoBoNT/A had developed partial or complete STF or had detectable titres of NABs [30]. The incidence of NAB formation per year was 0.37% in the entire cohort, which is well below all incidences of antibody formation reported for BoNT/A treatment so far. The “worst case”-estimation of the incidence of NAB formation under incoBoNT/A mono-therapy, which assumes induction of NAB on the two CD patients in the switch group during incoBoNT/A treatment, yielded a value of 0.41% which is below the low incidence of mouse lethality assay (MLA)-positive patients in onaBoNT/A long-term treated CD patients [31]. For this comparison, it should also be taken into account that the MHDA is at least fivefold more sensitive to NAB detection than MLA [25].

Yearly incidences of NAB formation under ona- or aboBoNT/A treatment are reported to be small (0.56–1.5% and 1.05–2.5%, respectively; for details see [22, 23]). In a large cohort of 212 onaBoNT/A or aboBoNT/A long-term treated CD patients, cross-sectional testing detected 31 MHDA-positive patients yielding an estimation of mean incidence of NAB induction in this cohort of 1.25% [22]. The prevalence of NABs after long-term treatment over 11.7 years was 14.6% [22]. In another, even larger cohort of 596 patients being treated with BoNT/A for different neurological indications, 83 MHDA-positive patients were detected by cross-sectional testing [23]. The prevalence of NABs after long-term treatment over 5.20 years was 13.9% and the mean incidence 2.68% per year [23].

Compared to these studies, the incidence of NAB induction in the entire cohort and the mono subgroup observed in this study was much lower. Therefore, the present study is in line with the hypothesis that NAB induction is dependent on the protein load of a BoNT/A preparation [21], since the protein load of the incoBoNT/A preparation (Xeomin®) is the lowest of all licensed BoNT/A preparations.

### Switching to incoBoNT/A in patients pre-treated with other BoNT/A preparations

This is also the first study on NAB formation and clinical effect in a larger cohort of patients who had unsatisfactorily been pre-treated with ona- and/or aboBoNT/A, but were then switched to BoNT/A. In the switch group, IMP was 61% and only slightly, but significantly (*p* < 0.01) lower than in the mono group (> 70%). This may result from the previous unsatisfactory experience with BoNT/A treatment. In the CD patients, initial TSUI was not significantly higher in the switch (9.0) than in the mono group (7.9). Mean improvement of TSUI score achieved by injection of 100 MU incoBoNT/A was (9.0–4.5/3.1 = 4.5/3.1 = 1.45) in the switch and close to (7.9–3.6 = 4.3/2.6 = 1.65) in the mono group. This indicates that incoBoNT/A may be as effective in switchers as in patients who were exclusively treated with incoBoNT/A. But because of the significant negative correlation of IMP with last TSUI (see Fig. 3b) which was slightly higher in the switch compared to the mono group, IMP was slightly lower in the switch than in the mono group.

In our switch group, two patients with CD were detected with a positive MHDA test and high paralysis times (> 130 mins). A similar case with prior treatment with complex protein-containing BoNT/A preparations, switched to incoBoNT/A and subsequent development of MHDA positivity has already been described [30]. Similar to our two patients, this selected patient had not been MHDA tested before the switch to incoBoNT/A. Therefore, it is uncertain if NABs developed under incoBoNT/A or had already been induced before the switch of therapy. But it is more likely that NABs had been induced during pre-treatment, since pre-treatment was unsatisfactory, NABs were probably induced early during the course of treatment [8] and NAB titres may decrease during continuous incoBoNT/A treatment over more than 3 years [24].

To clarify this uncertainty, further studies are recommended on patients who have developed immunoresistance to their first BoNT/A preparation and have been switched to a second one, which control the development of NAB formation and the clinical effect in parallel.

## Conclusions

In agreement with Aoki and Guyer [21], we conclude that: “Due to the association between neurotoxin (complex) protein load and neutralizing antibody formation, the optimal strategy (for BoNT/A therapy) would be to minimize the risk of developing neutralizing antibodies. This can be accomplished by treating patients with a neurotoxin preparation that contains the lowest possible amount of neurotoxin (complex) protein per effective dose” [21].

### Limitations of the study

The present study demonstrates the low incidence and prevalence of NAB formation under incoBoNT/A long-term treatment. The results would have been even more convincing if NABs had also been determined before the switch to incoBoNT/A in the switch group. Furthermore, for the present cross-sectional study, patients were selected according to the duration of pre-treatment and duration of the following incoBoNT/A therapy (see “Methods”). We would have appreciated if we could have analysed all patients who were on continuous incoBoNT/A therapy with or without pre-treatment. However, that would have exceeded the cost limits by far.
